# Identifying Hubs in Protein Interaction Networks

**DOI:** 10.1371/journal.pone.0005344

**Published:** 2009-04-28

**Authors:** Ravishankar R. Vallabhajosyula, Deboki Chakravarti, Samina Lutfeali, Animesh Ray, Alpan Raval

**Affiliations:** 1 Keck Graduate Institute of Applied Life Sciences, Claremont, California, United States of America; 2 California Institute of Technology, Pasadena, California, United States of America; 3 University of Chicago, Chicago, Illinois, United States of America; 4 School of Mathematical Sciences, Claremont Graduate University, Claremont, California, United States of America; Fondazione Telethon, Italy

## Abstract

**Background:**

In spite of the scale-free degree distribution that characterizes most protein interaction networks (PINs), it is common to define an *ad hoc* degree scale that defines “hub” proteins having special topological and functional significance. This raises the concern that some conclusions on the functional significance of proteins based on network properties may not be robust.

**Methodology:**

In this paper we present three objective methods to define hub proteins in PINs: one is a purely topological method and two others are based on gene expression and function. By applying these methods to four distinct PINs, we examine the extent of agreement among these methods and implications of these results on network construction.

**Conclusions:**

We find that the methods agree well for networks that contain a balance between error-free and unbiased interactions, indicating that the hub concept is meaningful for such networks.

## Introduction

A large number of cellular processes are mediated by physical interactions among proteins, including signal transduction, enzyme activity, and post-translational modification. The elucidation of large networks of protein-protein interactions has contributed to the identification of biochemical and signaling pathways, and to functional annotation of genes. Such networks have been systematically determined and explored in the baker's yeast *Saccharomyces cerevisiae* [1], [2], [3], [4], [5], [6], bacteria [7] and, more recently, in other organisms [8] such as the fruit fly [9] and the nematode worm [10] via a combination of high-throughput experimental methods, data mining, and computational predictions. One of the earliest observations relevant to the topology of a large protein-protein interaction network was that it possesses the “scale-free” property [11], i.e., the nodal degree distribution of the network is a power-law distribution [12], [13], or nearly so, and hence does not help identify any special degree “scale”. Even so it has been a common practice in the analysis of protein interaction networks to define an *ad hoc* threshold or degree scale such that all nodes (proteins) that have degree higher than this threshold are considered to be special in some sense and are called “hub” nodes. The notion of a hub protein is a compelling one because hub proteins, though defined arbitrarily, often do have special biological properties: they tend to be more essential than non-hub proteins [14], [15], they are found to play a central role in modular organization of the protein interaction network [16], [17], and some studies indicate that hub proteins may also be evolutionarily conserved to a larger extent than non-hubs [18]. However, at least partly because of the “scale-free” nature of protein interaction networks there is no consensus in the literature on the degree threshold that defines a hub. It is also unclear whether the biological significance of hubs is relatively insensitive to their precise definition. Examples of varying criteria used to define hubs include: in Batada et al. [19], the top 95% and 50% of the high degree nodes were defined as hubs in two different contexts; in Reguly et al. [20], the network was partitioned according to scale and in each sub-network hubs were identified as nodes with 95% of the connectivity; in Han et al. [17], nodes with degree greater than 5 were labeled as hubs; in Ekman et al. [21], nodes with degree greater than 8 were labeled as hubs; in [22], a degree cutoff of 20 was used to define hub proteins. In Jin et al. [23], the top 20% of proteins each with more than 12 partners were selected as hubs.

Given the largely *ad hoc* definition of hub proteins it is possible that many special properties attributed to hubs may be simply a consequence of the definition used. While this ambiguity may be alleviated by examining correlations between degree and other attributes without defining a category of nodes called hubs [24], [25], [26], it is still true that the notion of a special class of hub nodes remains ingrained in the literature without systematic analysis of whether it is reasonable to define this class. This work represents an attempt to carry out such an analysis.

The class of hub nodes in a protein interaction network may be defined by specifying, as stated above, a degree threshold such that all proteins with degree higher than this threshold are hubs or by specifying a number threshold such that when proteins are ranked by their degree a certain number of proteins from the top of this ranked list are hubs. In either case an objective definition of hub proteins will require criteria used to specify these thresholds that can be applied in the same manner to different networks.

What might these criteria be? Hub proteins (defined in an *ad hoc* fashion) are often found to have lower connectivity among themselves than non-hub proteins [27], [28]. Therefore, one way to define hub proteins could involve identifying the set of high-degree proteins that has significantly lower mutual connectivity than proteins that do not lie in this set. Whether such a definition is reasonable depends on whether proteins so identified have the biologically interesting properties that are usually attributed to hubs.

In contrast, it is also possible to define the set of hub proteins using the biological properties themselves. For example, it has been reported that the set of hub proteins in *Saccharomyces cerevisiae* may be divided into the so-called “date” and “party” hubs that are functionally quite distinct [19]. This division is in turn possible when the coexpression between hub proteins and their network neighbors follows a bimodal distribution. The bimodality implies the existence of two classes of hubs, one with low (albeit positive) averaged coexpression values and another with high averaged coexpression values [17]. Thus, another way to define hub proteins is to find the set of high degree proteins whose neighbor coexpression distribution is statistically significantly bimodal. If the set is not unique, we may more precisely define hub proteins as those whose neighbor coexpression distribution is maximally bimodal.

Yet another biologically important property that hub proteins are found to have is that they are significantly enriched for essential proteins [14]. Thus, a third way of defining the set of hub proteins is to identify the set of high degree proteins that is statistically significantly enriched for essential proteins as compared to non-hubs, perhaps maximally so.

The three criteria discussed above are not meant to represent an exhaustive set; rather, they represent three properties most commonly attributed to hubs: lack of intra-set connectivity, bimodality of coexpression, and enrichment for essentiality. As a way of examining the meaning (or lack thereof) of the hub concept, we apply hub definitions based on these three criteria to four differently constructed high confidence protein interaction networks in *Saccharomyces cerevisiae* (Methods), examine the extent to which the definitions agree, and report our results and interpretations thereof below.

## Methods

### Protein interaction and mRNA expression data

In order to test our criteria for defining hubs in protein interaction networks, we used four different multi-validated protein networks constructed for *S. cerevisiae*: the HC network (with 2998 nodes and 9258 edges) was obtained from Batada et al. [19]; the literature curated LC network (with 3307 nodes and 14169 edges) was obtained from Reguly et al. [20] by filtering the full literature curated dataset for protein-protein interactions and removing redundant edges; the FYI network (with 1379 nodes and 2493 edges) was obtained from Han et al. [17]; finally, the HC^h^ network (with 1787 nodes and 3004 edges) [19] was constructed from HC by selecting only those high-throughput interactions that were multi-validated by two or more different methods.

The coexpression criterion for defining hubs (Results) requires the use of gene expression data. Yeast mRNA expression data profiles corresponding to five different conditions [29] were used in this study: stress response [30] (174 data points), cell cycle [31] (77 data points), pheromone treatment [32] (56 data points), unfolded protein response [33] (10 data points) and sporulation [34] (9 data points), all of which were normalized and combined into a single dataset - the yeast compendium [17]. This compendium of 326 data points was constructed by combining the expression data for the five different conditions from [29] and normalizing the datasets such that data for each gene had zero mean and unit standard deviation across all experimental conditions. Missing data points were imputed by means of row averages for each gene. Finally, a list of essential genes in yeast (required for the essentiality criterion – see Results) was obtained from [35].

### Relative connectivity of degree-ordered subgraphs

One way to define hubs is to use the mutual connectivity properties of high degree nodes in protein interaction networks. It has been reported, for example, that hub-hub connections in PINs are suppressed [27], [28]. To define hubs using a mutual connectivity criterion, one may therefore construct a subgraph connectivity measure in the following way.

#### Relative connectivity of a subgraph

A simple measure of topological connectivity of a graph is the relative size of its largest component, i.e., the number of nodes in the largest component divided by the total number of nodes in the graph. We will call this measure the relative connectivity *f* of the graph. Clearly, when *f = 1*, the graph is topologically connected, while a graph with a small value of *f* must be composed of a large number of disconnected components or fragments.

**Figure 1:**
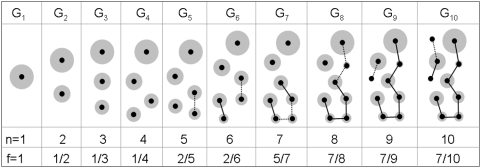
A cartoon illustrating relative connectivity of subgraphs. Successive subgraphs are generated from a ranked degree list, and the relative connectivity *f* is computed from them. Each node is represented by a black center with a gray ‘halo’ whose size is proportional to the degree of the node. Note that newer nodes have smaller halos (lower degrees). Interactions involving newly added nodes are shown as dotted edges, while previously established interactions are shown as dark edges. Note that all subgraphs upto *G_4_* are completely disconnected in this example.

Suppose we are now given a large network *G*, and we construct a ranked list of nodes ordered by their degree in decreasing order. We may extract from the original network the subgraph corresponding to the first *n* nodes in this ranked list, and call this subgraph *G_n_*. Note that, if hub-hub interactions are indeed suppressed, then the relative connectivity of *G_n_* should be small up to a certain value of *n* beyond which, as more and more “non-hubs” are included in *G_n_*, the relative connectivity should begin to increase, eventually reaching the relative connectivity of the entire network *G*. The process of constructing successive subgraphs of increasing size and computing their relative connectivities is illustrated in Figure 1. The key point here is that the value *n* at which the subgraph connectivity begins to rise could be interpreted as a natural boundary between “hub” nodes and “non-hub” nodes, thus leading to an objective characterization of what constitutes a hub node that is based purely on the topology of the network.

Note that other natural measures of relative connectivity are also possible. One example is a suitably normalized entropy of the distribution of component sizes. This entropy would be zero for connected networks and maximal for completely fragmented networks where every node is isolated. We found similar results when using this measure but ultimately chose the simpler definition of relative connectivity.

#### Statistical significance of a rapid increase in subgraph connectivity

We examined whether the boundary between hubs and non-hubs, i.e., the occurrence of a minimum followed by a rapid increase in the subgraph connectivity is a statistically significant feature. To assess this, we constructed, corresponding to each network studied, 10,000 random networks of the same size and degree sequence, following the configuration model of Newman [36]. The relative subgraph connectivity profiles were constructed for each of these randomized networks. The significance of an increase in a relative connectivity value *f_n_* for subgraph *G_n_* was then assessed by using the test statistic *s_n_* ≡ *f_n_*_+*k*_−*f_n_*. This statistic basically measures the increase in subgraph connectivity following the addition of the next *k* nodes to the subgraph. The corresponding P-value, for each *n*, was then empirically found as the fraction of random networks that had *s_n_* values larger than or equal to the observed *s_n_* value in the real network. The value of *k* is chosen heuristically: low values of *k* (close to 1) capture local fluctuations in the relative connectivity, whereas very large values of *k* capture gradual increases in the relative connectivity. In practice, we find that an intermediate value (*k* = 5) appears to delineate well the rapid increase in relative connectivity that characterizes the addition of non-hubs to a subgraph of hubs.

### Jensen-Shannon Divergence between distributions of essential gene composition

In order to assess the difference in composition of essential genes among hubs and non-hubs (see Results), a useful statistic is the value of the Jensen-Shannon divergence [37], [38], [39], [40] between two distributions *p_1_* and *p_2_*. This divergence is given by
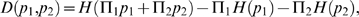
(1)where *H*(*p*) = −Σ_x_ *p*(*x*) log_2_ *p*(*x*) is the entropy of distribution *p*, and the weights Π_1_ and Π_2_ for the individual distributions are constrained to lie between 0 and 1 and to satisfy Π_1_+Π_2_ = 1. Here, we choose each weight to be proportional to the number of genes/proteins in the corresponding set, i.e., Π_1_ = *n/N* and Π_2_ = *(N*−*n)/N*, where *n* is the number of hubs, and *N* is the total number of nodes in the network.

## Results

### Hub definition based on relative connectivity of degree-ordered subgraphs

#### Relative subgraph connectivity profiles

**Figure 2:**
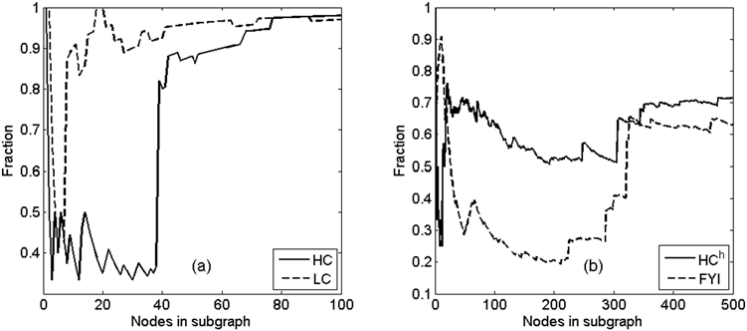
Relative subgraph connectivity as a function of number of nodes. The four yeast protein interaction networks studied are HC and LC (panel (a), first 100 nodes), HC^h^ and FYI (panel (b), first 500 nodes), showing regions of interest where the relative subgraph connectivity increases from a minimum.

The relative connectivity (Methods) for successive subgraphs *G_n_* was computed as a function of *n* for the four yeast protein interaction networks used in this paper. As shown in Figure 2, the relative subgraph connectivities in the HC^h^ and FYI networks reach a minimum around *n*≈200–300 nodes before beginning to increase and eventually approach the relative connectivity of the entire network. From the connectivity point of view, we may therefore identify roughly the first 200–300 nodes in these networks as hubs, since by themselves they form a highly fragmented subgraph. It is interesting to note that, for the FYI network, this hub definition agrees well with the *ad hoc* hub definition in [17] (see Table 1). In a similar way, the relative subgraph connectivity plots for HC and LC networks in Figure 2 show that roughly only the first 40 nodes in the HC network and the first 10 nodes in the LC network may be identified as hubs by the connectivity criterion.

**Table 1:** Comparison between degree cutoffs for defining hubs by our relative connectivity based method and definitions used in the literature.

Dataset	Reference	Nodes	Edges	Results based on relative connectivity		Hub definitions used in the literature	
				Degree cutoff	Number of hubs	Degree cutoff	Number of hubs
HC	[19]	2998	9258	33	40	16–21	150–300
LC	[19], [20]	3307	14169	85	11	82, 17	12; 294
HC^h^	[19]	1787	3004	17	20	7–10	90–180
FYI	[17]	1379	2493	5	300	5	320

#### Statistical significance of sharp increases in the subgraph connectivity

**Figure 3:**
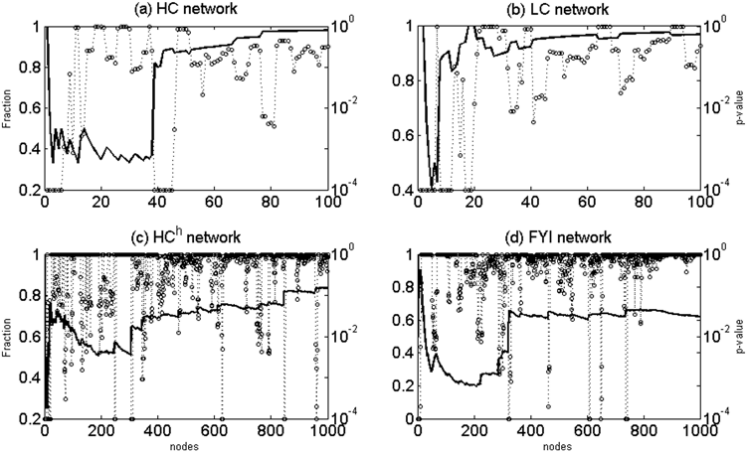
Statistical significance of relative subgraph connectivity. Empirical P-values (dashed lines) for significance of the relative connectivity measure (solid lines) for all the four networks were computed using 10,000 random networks corresponding to each real network. P-values that are less than 10^−4^ can be identified by the circles on the x-axis in each panel.

Next, the statistical significance of sharp increases within subgraph connectivity profiles was assessed, as described in Methods. The corresponding P-values were then plotted as a function of *n* for the four networks studied, with *k* = 5 (Figure 3). We indeed found that the regions of transition between low and high subgraph connectivity identified earlier are statistically significant (P-value<10^−4^), although other statistically significant regions corresponding to local fluctuations in subgraph connectivity were also identified (Figure 3). We find (data not shown) that the statistical significance of the transition regions persists for a broad range of values of *k* and therefore these regions correspond to robust features of the relative connectivity profiles. On the other hand, local fluctuations in the relative connectivity do not turn out to be as robust. Note that the presence of statistically significant sharp increases in relative subgraph connectivity shows that such sharp increases are not found in random scale-free networks, because in each case the real protein interaction network is compared to random scale-free networks with the same degree sequence.

We note that, although it has been reported that hub-hub interactions are not suppressed in the HC network [19], Figures 2 and 3 show that roughly the first 40 high degree nodes of this network form a statistically significant maximally fragmented subgraph (in the sense of relative connectivity), and therefore, hubs identified by the relative connectivity criterion by definition are sparsely connected among themselves. The overall qualitative nature of the relative connectivity profiles is also in agreement with the idea that hub proteins tend to have more interactions with non-hubs [27].

### Hub definition based on co-expression relationships between interacting proteins

Hub proteins have been reported to have special properties with respect to their level of co-expression with neighboring proteins in a protein interaction network. Han et al. [17] found, in the case of the FYI network, that the set of hubs could be further sub-divided into “date” and “party” hubs, where party hubs exhibit significantly higher coexpression with their protein interaction neighbors than date hubs. Party hubs were further found to lie within protein interaction modules, while date hubs connect modules. It is important to note, as later pointed out by Batada et al. [19], that the subdivision into date and party hubs is only possible if the distribution of correlation between the expression profiles of hubs and their interaction partners is *significantly* bimodal in nature, thus admitting two separate interpretations for the two peaks in the coexpression distribution. However, using a hub definition similar to that used in [17], as well as other hub definitions, Batada et al. [19] find that the coexpression distribution between hubs and their partners is not significantly bimodal, thus casting doubt upon the date and party hub distinction. This issue has been the topic of some recent controversy [41], [42], including attempts to resolve it by taking into consideration the network motifs of which the date and party hubs are part [23].

#### Bimodality of coexpression distribution when hubs are identified using the relative connectivity criterion

**Figure 4:**
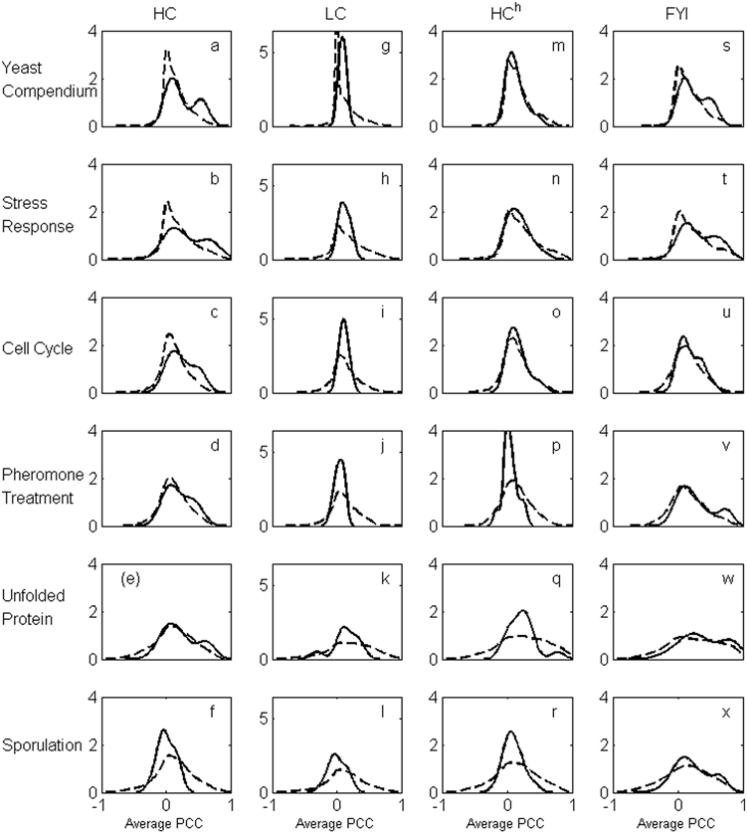
Expression correlation distributions. The panels display distributions of the average Pearson correlation coefficient (PCC) between expression profiles of hubs with their interaction partners (solid line) and non-hubs with their interaction partners (dashed line) for HC ((a)–(f)), LC ((g)–(l)), HC^h^ ((m)–(r)) and FYI ((s)–(x)). The set of hubs for each network was determined from the relative subgraph connectivity analysis. Average PCC values were computed using normalized gene expression profiles over the full yeast compendium that includes expression data under all five conditions each of which is also analyzed individually.

**Figure 5:**
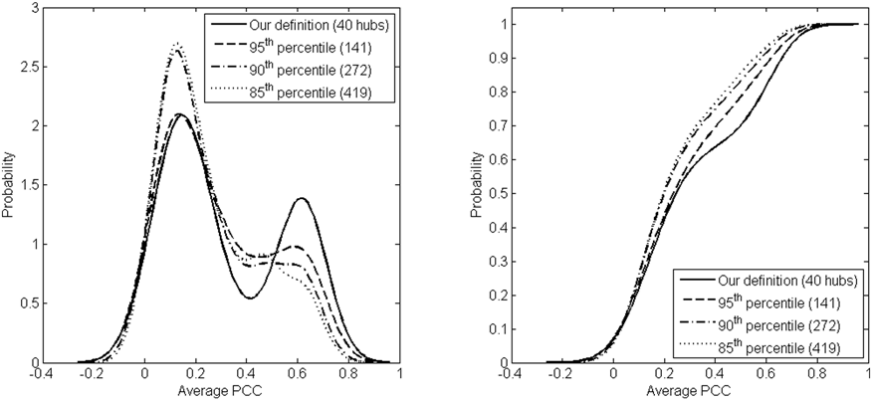
Bimodality of PCC distribution for the HC network. Inclusion of non-hub nodes into the list of HC hubs leads to reduction in bi-modality of the average PCC distribution. This can be seen as the number of hubs included increases from 40 to 419 in the HC dataset. The panel on the left displays smoothed probability density functions corresponding to the average PCC distribution while the panel on the right displays the cumulative distribution functions. Percentiles refer to the percentages of top high degree nodes included in the hub set, following [19].

In contrast to the approaches mentioned above, by following the connectivity profile analysis carried out in the previous subsection, if we identify only the top 40 or so high degree proteins as hubs in the HC network, it is apparent that they do exhibit a bimodal coexpression distribution with their protein interaction neighbors under several expression conditions (Figure 4, (a) to (f)). Furthermore, the reason why this bimodal distribution was not observed in the same network by Batada et al. [19] is because, in the absence of any objective criterion to define hubs, these authors included many more high degree nodes in the hub set than are suggested, for example, by the relative connectivity criterion. Inclusion of as many high degree nodes in the hub set as Batada et al. used leads to breakdown of bimodality in the coexpression distribution (Figure 5). It is remarkable that a hub set definition based on topology of the protein interaction network leads to a clear coexpression-based separation between party and date hubs. We may therefore explore the implications of defining a set of hubs based solely on the propensity of this set to significantly separate into date and party hubs. On the other hand, while the choice of hubs with the relative connectivity criterion does indicate significant bimodality in the coexpression distribution for HC and FYI networks, it is much weaker in the HC^h^ network and bimodality does not occur at all in the LC network. In the case of the LC network, this is most likely due to the fact that there are few nodes (according to the relative connectivity criterion) that can be treated as hubs, resulting in lack of significance due to small sample size.

#### Using significance of coexpression bimodality to determine number of hub nodes

To assess whether a given hub set can be significantly decomposed into party and date hubs, we make use, as in earlier work [19], of the dip test [43] for deviations from uni-modality of the PCC distribution. Rejection of the null hypothesis in this test will allow us to infer that there are at least two modes in the distribution, and that these two modes can be interpreted as corresponding to party and date hubs. It has been shown in [19] that the null hypothesis of uni-modularity can be rejected with 95% confidence (P-value≤0.05) provided the ‘dip’ statistic is at least as large as the value given by the formula from [19]

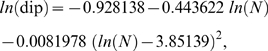
(2)where *N* is the size of the dataset from which the empirical distribution is obtained.

**Figure 6:**
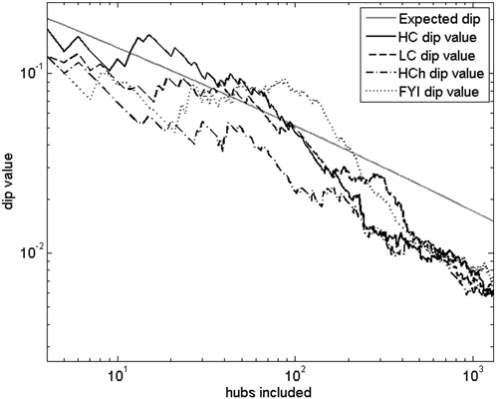
Dip statistics as a function of number of included hubs. Values of the dip statistic for all four networks studied as a function of the number of top degree nodes included in the hub set. The straight line marks the boundary between statistically significant and insignificant dip values (at 95% confidence).

As before, we ordered protein nodes in each of the four networks studied in decreasing order of degree and successively included more and more nodes in the hub set. For each constructed hub set, we computed the Pearson correlation coefficient (PCC) between hubs and their protein interaction neighbors, then computed the dip statistic for the PCC distribution. The result of this analysis is shown in Figure 6. Note that the HC network displays a statistically significant partitioning into date and party hubs if between about 15 and 90 of the top degree nodes are included in the hub set. Similarly, the FYI network admits such a decomposition if between about 50 and 200 top degree nodes are included in the hub set; the LC network admits the decomposition only if about the top 50 high degree nodes are included in the hub set; and finally, the HC^h^ network does not admit a date and party hub decomposition for any choice of the hub set.

Note that Han et al. [17] used about 200 hubs for the FYI network and yet appeared to find a bimodal distribution in PCC values, while we do not find significant bimodality when 200 or more hubs are included. This discrepancy could be attributed to the fact that we use 326 data points in the expression compendium as opposed to 315 used by Han et al. The additional 11 data points arise from including all 56 data points in the pheromone treatment dataset instead of only 45 data points used by Han et al. [17] (Methods).

### Hub definition based on composition of essential genes

It is well known that proteins with high degree in a protein interaction network are more likely to be essential than proteins of low degree [14], [19]. This observation may be used as yet another basis for defining a set of hubs: namely, as the set of high degree proteins that is statistically significantly more enriched for essential proteins as compared to proteins outside the set. More precisely, we again order protein nodes in decreasing order of degree, successively include a larger number of top degree nodes in the hub set, and examine the difference in composition of essential proteins between hubs and non-hubs as a function of number of nodes included in the hub set.

#### Measures of essential gene composition difference

**Figure 7:**
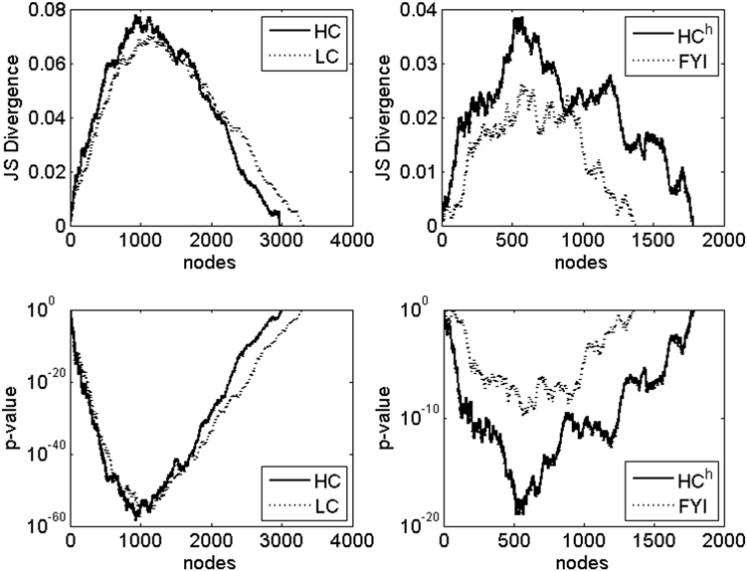
Essential gene enrichment. Enrichment for essential genes among hubs relative to non-hubs, as measured by the Jensen-Shannon divergence (upper panels) and the P-value for the Kolmogorov-Smirnov test (lower panels).

There are two natural measures of difference of composition of nodes of a certain type (here, essential proteins) between two sets of nodes (here, hubs and non-hubs). One well known measure is the P-value for the Kolmogorov-Smirnov test for difference in distributions. If *e_1_* is the fraction of essential proteins in the hub set and *e_2_* is the fraction of essential proteins among non-hubs, then the Kolmogorov-Smirnov test is a test for inequality between distributions *p_1_* ≡ { *e_1_*, 1−*e_1_*} and *p_2_* ≡ { *e_2_*, 1−*e_2_*}. Another measure is the Jensen-Shannon divergence between these distributions (Methods). Both these measures are plotted in Figure 7 as a function of number of high degree nodes included in the hub set. Indeed, we find that the two measures display qualitatively reciprocal behavior for all four networks of interest: the Jensen-Shannon divergence is high where the Kolmogorov-Smirnov P-value is low, and vice versa.

#### Number of nodes to be included in the hub set for statistical significance of difference in composition of essential genes

It is clear that a hub definition based on statistical significance of the compositional differences of essential proteins among hubs and non-hubs is far more unconstrained than the previous two hub definitions we have considered: most choices of hub sets, that is, choosing between the top 3 and 2973 high degree nodes for HC (99.01% of nodes); 15 and 3240 for LC (97.52%); 17 and 1756 for HC^h^ (97.31%); and 138 and 1232 (79.33%) for FYI, as hub nodes leads to statistically significant (P-value≤0.05) compositional differences of essential genes, with hubs having significantly more essential genes than non-hubs. Specifically, except for the LC network, relative connectivity based hub definitions and coexpression based ones are consistent with statistically significant essential gene compositional differences, although none of the definitions correspond to a maximally significant compositional difference (this maximum occurs when approximately 900 top degree nodes are included in the hub set for the HC network, 1200 for LC, 550 for HC^h^, and 550 for FYI). For the LC network, the connectivity based hub definition and the essentiality based one do not quite overlap: by the connectivity criterion, only about the top 10 high degree nodes can be included in the hub set, whereas by the essentiality criterion, at least 15 of the top high degree nodes must be identified as hubs for a statistically significant difference in number of essential genes among hubs versus non-hubs. However, given errors and incompleteness in protein interaction network data, these numbers appear close enough to pronounce weak agreement between the connectivity-based and essentiality-based criteria for defining the LC hub set. We address this and related issues in greater detail in the Discussion.

### Robustness of hub definitions

The four yeast protein interaction networks that we examine, although high confidence, are still subject to some error. Specifically, it is reasonable to expect that the false negative rates for these networks could be quite high, although the false positive rates are low. In such situations, it is useful to work with clean, simulated data in order to test the applicability of a new concept or algorithm (for example, reverse engineering algorithms for gene regulatory networks can be tested using simulated gene expression data [44]). However, due to the lack of availability of simulated protein interaction networks where the nodes have a clear functional meaning, we instead accounted for errors in protein interaction data by randomly adding and removing edges from the parent protein interaction networks (this addresses both false positives and false negatives) and examining the resulting change in hub definitions.

**Figure 8:**
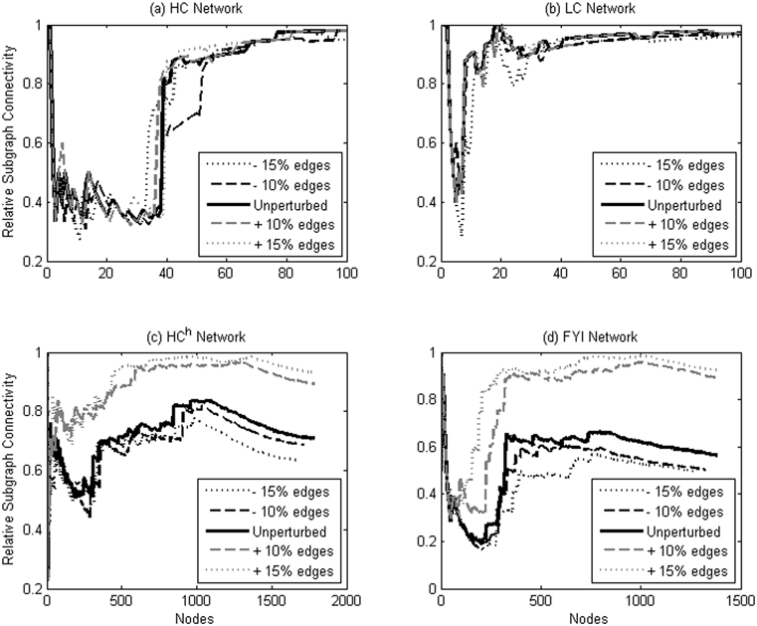
Robustness of relative subgraph connectivity. Relative subgraph connectivity profiles for unperturbed versions of all four networks are shown, along with the corresponding profiles upon random addition and removal of 10% and 15% of the edges in the unperturbed networks.

Figure 8 displays the effects of random edge addition and deletion on the relative subgraph connectivity profiles. For each of the four networks studied, 10% and 15% of the edges present in the network were added at random, and the same number of edges removed at random, respectively. In each case, the overall shape of the subgraph connectivity profiles remains similar to the one in the unperturbed case. For HC and LC, we find that the location of the sharp increase in subgraph connectivity does not appreciably change upon random addition and removal of up to 15% of edges. For HC^h^ and FYI, there is similarly no appreciable change to the subgraph connectivity profiles upon random removal of up to 15% of edges but the profiles do change upon random addition of edges. These two networks contain far fewer edges in comparison to the other two networks: HC^h^ and FYI contain about a third of the number of edges in HC and about a fifth of the number of edges in LC. It is therefore expected that HC^h^ and FYI would have a large false negative rate, and therefore that addition of edges would reduce the false negative rate and substantially change the connectivity profiles. It is also expected that addition of edges would bring the connectivity profiles of these two networks closer to that of LC and HC, as observed. Furthermore, we find that change in the location of the sharp rise in relative connectivity does not substantially affect the degree value at which that rise occurs, even though it affects the number of nodes classified as hubs by the connectivity criterion: the degree cutoff value changes from 17 (unperturbed value) to 16 (15% edges added) for HC^h^, and from 5 (unperturbed value) to 7 (15% edges added) for FYI. We thus find that the connectivity-based criterion for hubs is reasonably robust with respect to random edge deletion and addition. Furthermore, we found that the other two criteria are extremely robust: random addition and deletion of up to 15% of the edges has no appreciable effect on the statistically significant ranges of cutoff values for all four networks (data not shown).

## Discussion

Our aim in this work was to examine objective criteria that could be used to define hubs in protein interaction networks. We presented three such criteria here - one based purely on network topology and the other two involving gene expression and function. We applied these criteria to four differently constructed protein interaction networks in *S. cerevisiae*. Our results lead to some observations regarding network topology, the role of hubs, and gene/protein function.

First, we found that all four networks displayed a characteristic and relatively sharp statistically significant increase in relative subgraph connectivity as successive lower degree nodes are added to the subgraph. This increase identifies a clear “scale” in these power law networks, and marks a transition between high degree nodes (hubs) and intermediate degree nodes such that these two classes have very different topological properties: hubs by themselves form a highly fragmented subgraph while intermediate degree nodes play the role of mediating connections among hubs so that the subgraph formed by hubs plus intermediate degree nodes has high connectivity.

Second, we found that, for two networks – namely, FYI and HC, the hub notion as defined by this transition agrees well with the hub notion defined by the ability to split the hub set into date and party hubs based on their neighbor coexpression characteristics. In the process, we also found that the split between date and party hubs is quite sensitive to how hubs are defined in the first place, an issue that has largely been ignored in recent controversies regarding the separation of date and party hubs. We also found no agreement between the connectivity based hub notion and the expression based one for the HC^h^ and LC networks. We note that both FYI and HC networks are constructed by combining literature-curated and high-throughput data so that the resulting network is, to a large extent, balanced in terms of both bias and error. However, HC^h^ is most likely error-prone (although more unbiased than FYI or HC) and LC is most likely biased (although more error-free than FYI or LC). It is intriguing that two very different objective criteria for defining hubs agree well for networks that have a balance of error-free and unbiased interactions. Third, we find, in all four networks, that virtually any characterization of the hub set results in a significant difference in essential gene composition among hubs versus non-hubs. This is of course a result of previously reported strong correlations between degree and essentiality, and it implies that statistical significance of difference in essential gene composition is not a very precise way to define hubs in protein interaction networks.

To summarize, it appears that the hub concept is more meaningful for “balanced” networks (because of the agreement between three independent notions of a hub) than it is for networks that are dominated by error-prone, high-throughput data or networks that are compendia of error-free but biased literature-curated interactions. This observation, coupled with the methods presented here, could therefore be used both to test a protein interaction network constructed by a combination of methods as well as to define hub proteins in such a network.

Finally, we remark that our methods can be generalized beyond the simple notion of degree centrality to other more complicated centrality measures that also have functional significance. Just as the sharp rise in connectivity at a certain degree defines a degree “scale” that can be used to differentiate hubs from non-hubs, other centrality measures could possess characteristic scales in protein interaction networks.
